# Reliability of a novel serious game using dual-task gait profiles to early characterize aMCI

**DOI:** 10.3389/fnagi.2015.00050

**Published:** 2015-04-22

**Authors:** Ioannis Tarnanas, Sotirios Papagiannopoulos, Dimitris Kazis, Mark Wiederhold, Brenda Widerhold, Magda Tsolaki

**Affiliations:** ^1^3rd Department of Neurology, Medical School, Aristotle University of ThessalonikiThessaloniki, Greece; ^2^Division of Cognitive and Restorative Neurology, Virtual Reality Medical CenterSan Diego, CA, USA; ^3^Virtual Reality Medical Institute, Brussels Life Sciences Incubator, Catholic University’s Woluwe CampusBrussels, Belgium

**Keywords:** mild cognitive impairment, early diagnosis, motor performance, virtual reality, test–retest reliability, Alzheimer’s disease

## Abstract

**Background:** As the population of older adults is growing, the interest in a simple way to detect characterize amnestic mild cognitive impairment (aMCI), a prodromal stage of Alzheimer’s disease (AD), is becoming increasingly important. Serious game (SG) -based cognitive and motor performance profiles while performing everyday activities and dual-task walking (DTW) “motor signatures” are two very promising markers that can be detected in predementia states. We aim to compare the consistency, or conformity, of measurements made by a custom SG with DTW (NAV), a SG without DTW (DOT), neuropsychological measures and genotyping as markers for early detection of aMCI.

**Methods:** The study population included three groups: early AD (*n* = 86), aMCI (*n* = 65), and healthy control subjects (*n* = 76), who completed the custom SG tasks in three separate sessions over a 3-month period. Outcome measures were neuropsychological data across-domain and within-domain intra-individual variability (IIV) and DOT and NAV latency-based and accuracy-based IIV. IIV reflects a transient, within-person change in behavioral performance, either during different cognitive domains (across-domain) or within the same domain (within-domain). Test–retest reliability of the DOT and NAV markers were assessed using an intraclass correlation (ICC) analysis.

**Results:** Results indicated that performance data, such as the NAV latency-based and accuracy-based IIV, during the task displayed greater reliability across sessions compared to DOT. During the NAV task-engagement, the executive function, planning, and motor performance profiles exhibited moderate to good reliability (ICC = 0.6–0.8), while during DOT, executive function and spatial memory accuracy profiles exhibited fair to moderate reliability (ICC = 0.3–0.6). Additionally, reliability across tasks was more stable when three sessions were used in the ICC calculation relative to two sessions.

**Discussion:** Our findings suggest that “motor signature” data during the NAV tasks were a more reliable marker for early diagnosis of aMCI than DOT. This result accentuates the importance of utilizing motor performance data as a metric for aMCI populations where memory decline is often the behavioral outcome of interest. In conclusion, custom SG with DTW performance data provide an ecological and reliable approach for cognitive assessment across multiple sessions and thus can be used as a useful tool for tracking longitudinal change in observational and interventional studies on aMCI.

## Introduction

Even though a number of risk factors for sporadic Alzheimer’s disease (AD), the most common type of dementia, have been discussed (e.g., diagnosis of mild cognitive impairment (MCI), hippocampal atrophy, family history of AD, apolipoprotein-E ε4 allele [APOE-ε4]), one of the most well-documented risk factors for the disease is increasing age (Ferreira et al., 2014; Naj et al., 2014). Neurodegenerative changes such as atrophy, which is characteristic of AD, and occasionally of other dementing diseases such as fronto-temporal lobar dementia (FTLD) or Hippocampal Sclerosis, have a relatively long pre-morbid asymptomatic period (Lindberg et al., 2012a; Pelletier et al., 2013). At the same time, despite the fact that no cognitive symptoms may be obvious during the pre-morbid period, by the time AD is diagnosed, sufficient neuronal injury has occurred such that reversal of the disease is perhaps unlikely (Petersen, 2003; Lindberg et al., 2012b; Lockhart et al., 2012). This has therefore raised considerable interest in the prodromal stage of AD, involving revised criteria for diagnosing an early clinical stage of AD (“MCI due to AD,” aMCI or MCI-AD; Dubois and Albert, 2004; Michon, 2009; Sperling et al., 2011) and “Prodromal AD”(Albert et al., 2011) and incorporating biomarkers to increase the certainty of the diagnosis (Dickerson et al., 2013).

The accuracy of early diagnosis for dementia is increasingly important for both therapeutic and scientific investigations. Many of the early-onset dementias are treatable, and the presentation of the common degenerative diseases of late life, such as AD, can be different when presenting in the fifth or sixth decade. The currently available diagnostic tests have moved the field closer to early diagnosis of AD; however, differential diagnosis is broad, and a definitive diagnosis is made only with the development of clinical dementia and the presence of amyloid plaques and neurofibrillary tangles at autopsy (Snowden et al., 2011). An ideal AD biomarker should be able to satisfy the following criteria: the ability to diagnose AD with high sensitivity and specificity as confirmed by the gold standard of autopsy validation, detect early-stage disease, and track the progression of AD and monitor disease progression or therapeutic efficacy (McKhann et al., 2011). This understanding could offer the potential for tailored treatments and a specific diagnosis for both early-onset and late-onset dementia. MCI-AD, or aMCI, is a term used to describe early AD signs that precede functional and cognitive impairment (Sperling et al., 2011) and may be clinically indistinguishable from what is described as “probable AD” (Albert et al., 2011; Dickerson et al., 2013). Epidemiological studies have suggested that the most common form of aMCI is a multiple deficit syndrome with memory impairment and a 10–15% annual risk of conversion to AD (Portet et al., 2006). According to some recent studies, MCI individuals with amnestic syndrome of the hippocampal type (HaMCI), compared to those with the amnestic syndrome of the non-hippocampal type (NHaMCI), are the leading at-risk subgroup of the MCI population for the development of dementia due to AD (Sarazin et al., 2007). However, it is still controversial whether the tests designed to detect hippocampal amnestic syndrome (Duara et al., 2013), such as atrophy of the hippocampus in the CA1 subfield region (Burger, 2010; Fletcher et al., 2014), are superior to other tests for the detection of early-stage dementia (Albert et al., 2011; McKhann et al., 2011). Yet, in addition to the atrophy of hippocampus only in the CA1 subfield region experts recently developed the harmonized protocol for the manual segmentation of the whole hippocampus (Prestia et al., 2013, Alzheimers Dementia). Indeed, after a harmonization effort lasted 4 years and funded by the Alzheimer’s Association, world experts converged onto the harmonized segmentation protocol, which. the European Medicines Agency has qualified as an enrichment biomarker to enroll mild and moderate as well as predemented AD subjects in regulatory clinical trials.

Besides the usefulness of cued recall as a diagnostic tool for aMCI and AD (De Jager et al., 2010; Kelemen and Fenton, 2010; Carlesimo et al., 2011; Chechko et al., 2014), emerging evidence has demonstrated the value of walking stability and variability analysis as an early indicator of aMCI and AD (Persad et al., 2008; Gillain et al., 2009; Theill et al., 2011; Montero-Odasso et al., 2012; Muir et al., 2012). More specifically, prospective studies over periods of five and 6 years in cohorts of 427 and 603 older subjects over 70 years of age demonstrated that initial quantitative measures of gait, such as velocity, variability, and frequency can predict the risk of developing dementia (Waite et al., 2005; Verghese et al., 2007; Montero-Odasso et al., 2014). However, the relationship between cognitive function in everyday abilities and gait variables in conditions other than normal walking (NW) is insufficiently understood in people with aMCI. In everyday activities, there are numerous dual-task walking (DTW) situations that require active involvement of the visual system (Al-Yahya et al., 2011). Observing how people walk while they perform a secondary task with a high demand on attention, i.e., a dual-task paradigm, has been used to assess interactions between cognition and gait. Executive function is often implicated in DTW because subjects must walk and adapt to new and/or complex situations that involve working memory, mental inhibition, and mental flexibility. In addition, less efficient executive functions in older adults have shown significant contribution to impairments in spatial memory, especially when spatial interference is high (Holden and Gilbert, 2012).

Another concept that has recently attracted the attention of researchers and clinicians is that of cognitive frailty. Recent work has defined cognitive frailty as a multi-dimensional geriatric syndrome characterized by the simultaneous presence of both physical frailty and cognitive impairment without the presence of a concomitant neurological disease (see Kelaiditi et al., 2013 for a review). Cognitive frailty is viewed as a potential precursor of neurodegenerative processes (Duron et al., 2014). However, few studies have made the link between motor fragility reflected by a reduction in walking velocity and cognitive fragility reflected by an early alteration in executive function and have hypothesized that alteration in motor performance could occur before detection of cognitive impairment (Perrochon et al., 2013).

In a similar vein, recent studies have shown that increased cognitive intra-individual variability (IIV) across accuracy scores from neuropsychological tests, representing different cognitive domains (across-domain IIV), might serve as a biomarker of cognitive frailty occurring before detection of prodromal AD (Kälin et al., 2014). More particularly, latency- (variability across response time performance scores) and accuracy-based IIV (variability across accuracy scores – correct vs. wrong responses) have reportedly been associated with functional decline (Dixon et al., 2007; Morgan et al., 2012), incident dementia (Holtzer et al., 2008), and probable AD (Brewster et al., 2012). A more recent study compared within- and across-domain IIV and APOE genotype between healthy control subjects (HCS), MCI, and AD in a single comparative study and found that within-domain IIV may constitute a cognitive marker for the detection of prodromal AD at the MCI stage, whereas across-domain IIV may detect beginning AD at the MCI stage (Kälin et al., 2014).

In this context, computerized cognitive assessments, such as serious games (SGs), can ideally be applied to detect subtle changes in both DTW and IIV between HCS and aMCI performance profiles (Robert et al., 2014). There is already evidence that SG can be successfully employed for the characterization of episodic and prospective memory profiles in MCI and AD (Werner et al., 2009; Weniger et al., 2011; Plancher et al., 2012) or even early screening for aMCI (Tarnanas et al., 2013, 2014). According to the literature, SG interactions require coordination of information by eliciting medium to high cognitive control, such as inhibition of external stimuli or processing speed (e.g., reaction time at interactive events), which is believed to be affected by aging (De Lillo and James, 2012; Korsch et al., 2014). Very recently, an innovative DTW concept for detecting cognitive impairment eliciting medium to high cognitive control, called the Walking Stroop Carpet, was demonstrated by Perrochon et al. (2013), but to our knowledge, this is the first study reporting on a complex everyday activities SG employing DTW, which might indicate prodromal AD.

The aim of this study was to (1) systematically evaluate the reliability of two SGs—a high cognitive control, requiring inhibition of external stimuli, and planning a virtual day-out task without DTW (DOT) and a high cognitive control, requiring inhibition, navigation task with DTW (NAV)—and (2) explore the stability of test–retest measurements as a factor of the number of SG sessions.

## Materials and Methods

### Participants

A total of 270 participants (HCS *n* = 100, aMCI *n* = 80, and AD *n* = 90) were considered for analysis from ongoing studies at the 3^rd^ Neurological Clinic of the Aristotle University of Thessaloniki, Greece and from the Greek Association for Alzheimer Disease and Related Disorders (GAADRDs) Memory Clinics, belonging to the 3^rd^ Neurological Clinic of the Aristotle University of Thessaloniki. The study was carried out in accordance with the Declaration of Helsinki, and the participants were recruited from the outpatient population of the GAADRD Memory Clinics or by advertisement in the local media. All subjects had complete cognitive baseline data acquired between January 2010 and April 2013 with written informed consent obtained prior to study participation. From the original HCS sample, 24 subjects were excluded from the analyses due to alcohol abuse (*n* = 2), dropout (*n* = 6), and medication (*n* = 16). Also, 15 aMCI and six early AD subjects were also excluded due to dropout after the initial assessment. Thus, a total of 76 HCS, 65 aMCI, and 86 early AD patients were eligible for the analyses.

Amnestic MCI was diagnosed according to the criteria of Winblad et al. (2004), and all diagnoses were made by a multidisciplinary team under the supervision of an experienced psychiatrist. The diagnosis was made if the patient met the following criteria: (1) memory complaint, (2) abnormal memory for age, (3) normal activities of daily living, (4) normal general cognitive function, and (5) not demented. Structural magnetic resonance imaging (MRI) data were also available in our aMCI cases in order to exclude other conspicuous brain abnormalities that could account for cognitive decline. The diagnosis of AD was done according to the International Working Group (IWG-2) criteria considering three main markers: abnormal neuropsychological assessment, medial temporal atrophy on MRI, and abnormal Abeta42 or tau protein concentrations in the CSF (Dubois et al., 2014). Additionally, all three groups (HC, aMCI, and early AD) were screened for disorders, which could potentially produce cognitive impairment, i.e., depression; psychiatric, neurological, and other diseases. Such subjects were excluded if there were such disorders or medication use potentially affecting cognition. Furthermore, it was ascertained that all participants had normal or corrected to normal vision. All participants but three older adults were right-handed, according to the Edinburgh Handedness Inventory (Oldfield, 1971).

### Neuropsychological and Psychomotor Examination

All subjects were assessed with a standardized neuropsychological test battery. The neuropsychological test battery consisted of multiple tests covering the following cognitive domains: working and episodic memory, executive function, attention/psychomotor processing speed, language, and visual-constructive abilities. The Mini–Mental State Examination (Folstein, 1975) was used to assess global cognitive functioning. For episodic memory, the Grober–Buschke scale was used (Grober et al., 1988; Carlesimo et al., 2011). Short-term memory and working memory were investigated using a digit span forward test (Ramsay and Reynolds, 1995). Tests of executive functioning included verbal fluency and category fluency (the Set Test; Isaacs and Akhtar, 1972), Stroop (Stirling, 1979), and the TMT B (Tombaugh, 2004). Long term memory was assessed with the Rey Auditory Verbal Learning Test (RAVLT; Rosenberg et al., 1984). We determined impairment if at least one score per domain was 1.5 SD below group means compared to test-specific normative data.

In addition to the neuropsychological examination, participants also completed a baseline psychomotor evaluation in order to exclude physical frailty or other forms of physical disability that might affect the reliability of both DTW and across-domain and within-domain latency- and accuracy-based IIV across SG performance profiles, such as arthritis. The psychomotor examination included a number of simple and complex measures addressing the ability to understand and perform with accuracy specific physical performance tasks. These tasks included the following: (a) “Gait-speed” was measured as the time (to 0.1 s) required for a participant to walk a 4.6-m course at his or her usual pace after starting from a standstill and recorded by stopwatch, and b) the “Finger-Tapping Test” measured both the dominant and non-dominant hand using a computerized screening test, which measured finger tapping speed for a given duration of 10 s.

### Quantitative Gait Assessment

We performed a baseline motor evaluation of single-task spontaneous walking for 10 m in a normal environment on an 8-m electronic walkway (GAITRite®;, CIR Systems Inc, Sparta, NJ, USA). This tool is equipped with a portable, pressure-sensitive electronic walkway [793 cm × 61 cm × 0.6 cm (L × W × H)] which provides data for both spatial and temporal gait parameters. The simple-task trial consisted of walking the length of the mat at the participant’s usual pace. For the dual-task trials, participants walked at their usual pace, with no instruction to prioritize the gait or cognitive task, while doing the following cognitive tasks aloud: (i) counting backward from 100 by ones and (ii) naming animals. To balance and minimize the effects of learning and fatigue, the order of the dual tasks was randomized. Allowing both gait and cognitive tasks to vary provides a better representation of daily living activities, and the reliability of this protocol in people with MCI has been previously established (Montero-Odasso et al., 2009).

### SG Hardware Setup

**FIGURE 1:**
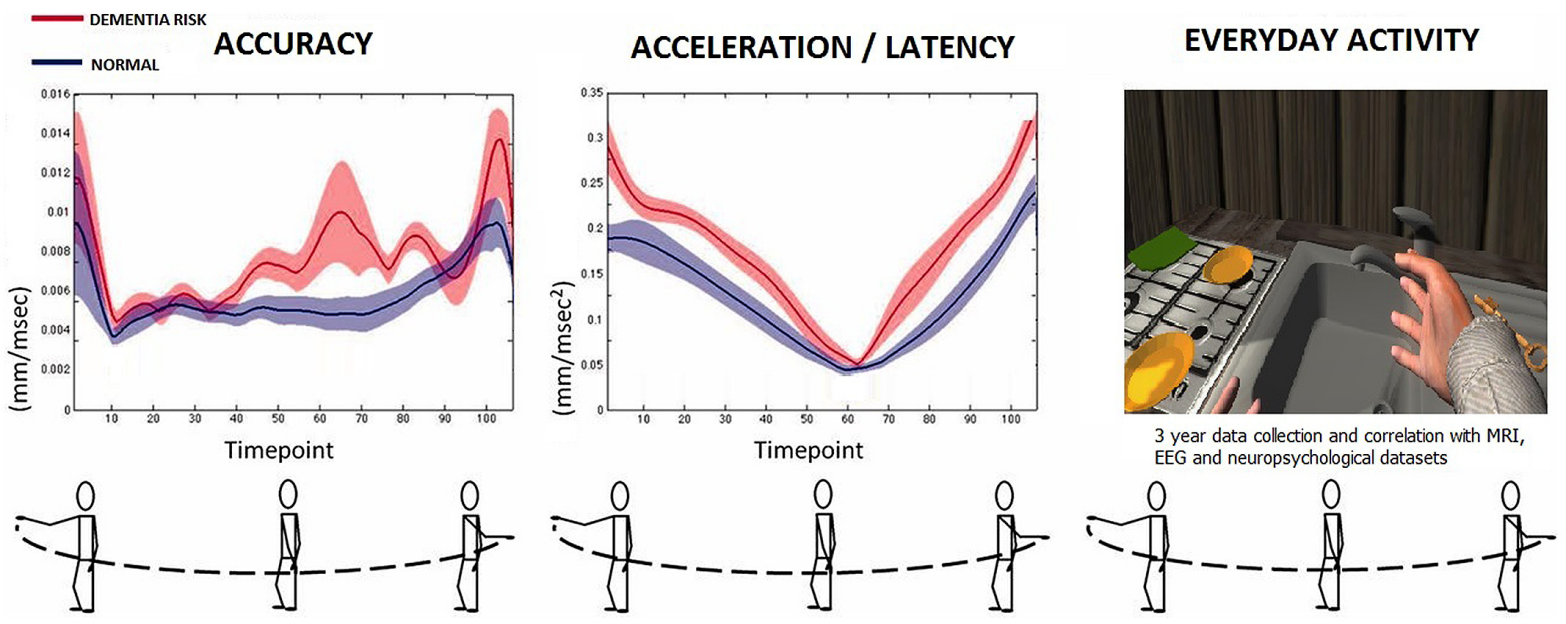
The custom SG with DTW setup.

The SG used is patent pending (XtremeVRI AG, Winterthur, Switzerland) and used mobile phone based Augmented Reality (AR) in order to present cognitive tasks and record all behavioral and kinetic responses while the subject navigated the AR environment inside his house (**Figure 1**).

### DOT and NAV Tasks

The novel SG used in this study consisted of two modules simulating complex activities of daily living (CADL): the 3D immersive reality day-out complex task without DTW (DOT) and the 3D immersive reality spatial NAV. The DOT was a complex task breakdown followed by a rehearsal exercise of a virtual apartment building fire evacuation drill. The drill included six different scenarios of increasing difficulty, where participants navigated the virtual environment using a first-person perspective and simple hand pointing gestures for forward, backward, and left, and right lateral movement, respectively. They could also use natural finger pointing and grabbing gestures in order to select, pick, drop, and move objects inside the virtual environment and had to complete each within 8 min. All participant movement within the virtual building was recorded at 10 Hz and represented as a series of *x*, *y*, *z* coordinates, with actions annotated and time stamped.

The DOT naturalistic actions script was based on an ordered list of right and wrong actions that was prepared by an occupational psychologist and was used to examine executive function and prospective memory as well as planning and reasoning in a complex emergency routine. The fire evacuation drill setting had six different simulated fire situations (from easy to more difficult) taking place at a virtual apartment block with three levels and five apartments per level. The task put a medium to high load on the cognitive control processes with which older adults prioritize, organize, initiate, and complete a number of subroutines (e.g., pick-up the phone and call the fire department, sound the fire-alarm) in order to evacuate safely and in the fastest possible way from an apartment level (e.g., second floor) to the ground area (e.g., determine and gather information on the size of the fire, avoid smoke, avoid wrong actions like using the elevator). In this sense, DOT is a complex activity of daily living, which previous research showed is a valid and reliable indicator of cognitive decline in elderly persons (Tarnanas et al., 2013).

The NAV task took place at the same virtual apartment block but with the player challenged in different aspects of executive function, such as volition, self-awareness, planning, inhibition of dominant response, and external distraction during response control, and dual-task coordination. The goal at difficulty level 1 was to navigate from point A to point B, after the route was demonstrated by a first-person perspective camera walkthrough without iteration. The NAV task took place with six levels of difficulty, with the addition of one more point of destination per difficulty level—for example, level 3 has three points to reach, level 4 has four, etc. Each level had a starting position (start) and an end position (goal) and multiple ways to arrive from start to goal. Participants were asked to make their way from start to goal in the shortest time possible. The NAV task placed a medium to high demand on higher order cognitive control processes, such as following a mental strategy to reach the goal with performance monitoring while inhibiting environmental stressors, such as virtual characters forcing the player to choose a less familiar route or interact with distractors in the virtual environment, a process which typically involves cognitive control.

According to the literature, interactions such as the DOT and NAV tasks, require participants to follow a mental strategy and monitor their performance by eliciting medium to high cognitive control, such as inhibition of external stimuli or processing speed (e.g., reaction time at interactive events; Kelemen and Fenton, 2010). This coordination of information to select appropriate behavioral responses is believed to be affected by aging (Korsch et al., 2014).

The order of participating in either the DOT or NAV tasks was random, and both started after each participant had 5 min to read written instructions detailing the task, virtual building layout, and task rules. Then, participants practiced the virtual environment using gestures to move around the building and completed 3 practice runs involving object collection, button pressing, unlocking the stairwell door with a key code, and folder sorting. This also allowed participants to familiarize themselves with the building. None of the practice runs were used in the main task. The practice session took in total approximately 20 min.

Participants played all difficulty levels of DOT and NAV in a baseline session (Visit 1), again all levels at a 1-month post-baseline session (Visit 2), and finally all levels at a 3-month post-baseline session (Visit 3). A total of three sessions were measured in order to assess test–retest reliability.

### Computation of IIV of DOT and NAV Performance

In order to apply IIV computations to the DOT and NAV performance profile data, we first had to categorize the performance profiles into accuracy-based and latency-based data. Since NAV was a more complex task than DOT, we conducted a principal component analysis (PCA) of participants’ performance data on the cognitive variables at their initial visit in order to create composite IIV measures. We then used the Spearman’s correlations to identify three accuracy- based and three latency-based performance categories, explained bellow, which place medium to high demands on higher order cognitive abilities for both DOT and NAV. Across-domain IIV was calculated with tasks representing different cognitive domains, while within-domain IIV was calculated with tasks representing cognitive control. Additionally, in order to avoid ceiling or floor effects, we added performances from each difficulty level and calculated a total performance profile from all difficulty levels per cognitive domain and category above in order to prevent suppressing variation at the extreme ends of the distribution.

To calculate DOT across-domain accuracy-based IIV, we used accuracy scores from three data categories, each representing a different cognitive domain: (1) spatial memory accuracy measured as the correct route selection, such as the nearest emergency exit or route for the evacuation of the virtual apartment building, according to the memorized virtual building layout; (2) planning accuracy measured as the correct order of subroutines execution, such as first sounding the fire alarm and then calling the fire department; and (3) executive functions accuracy measured as successful subroutines completion, such as sounding the fire alarm.

NAV across-domain accuracy-based IIV was calculated using accuracy scores from three data categories, each representing a different cognitive domain: (1) spatial memory accuracy measured as correct route selection, such as the nearest route for navigating from start to goal, according to the memorized virtual building layout; (2) planning accuracy measured as the correct order of subroutines execution, such as first going to point B and then to point C before goal; and (3) executive functions accuracy measured as successful subroutines completion, such as reaching each point from start to goal.

For calculating DOT within-domain accuracy-based IIV, we used accuracy performance data from three categories, each representing executive functions and eliciting recruitment of cognitive control processes. We calculated: (1) omissions of the subroutines, (2) repetition of the same subroutine, and (3) perseverations of incorrect order while performing the subroutines before completing the given script per difficulty level. According to the literature, virtual reality-based navigation, and interactions, such as the DOT task process, require participants to generate, maintain, and monitor a plan and to select and establish specific responses—therefore, accessing cognitive control (Cushman et al., 2008).

Accordingly, for calculating NAV within-domain accuracy-based IIV, we used accuracy performance data from: (1) omissions of the destination points between start and goal, (2) repetition of the same destination point, and (3) perseverations of incorrect order while navigating to the goal before completing the given script per difficulty level. Participants needed to maintain a goal while inhibiting a routine response in favor of a less familiar one, a process which typically involves cognitive control (West et al., 2002).

The DOT across-domain latency-based IIV was calculated using performance data from the participant’s timed response within three data categories, each representing a different cognitive domain: (1) total time to complete the navigation route per difficulty level, according to the memorized virtual building layout; (2) total time to complete the order of emergency evacuation subroutines execution; and (3) time of execution through acceleration data, such as “fast hand pointing gestures,” per subroutine completion.

In order to calculate the NAV across-domain latency-based IIV, we used performance data from the participant’s timed response at: (1) total time to complete the navigation route per difficulty level; (2) gait frequency at interactive events, such as avoidance of moving obstacles and distractors; and (3) gait parameters such as stride length, distance, and variability of stride while completing the navigation per destination point.

DOT within-domain latency-based IIV was calculated using timed response performance data from the following categories, eliciting recruitment of cognitive control processes: (1) reaction time of “navigation gestures” usage, measured as the time elapsed between the virtual character idle state and the next immediate “direction command”; (2) reaction time of “interaction gestures” usage, measured as the time elapsed between the virtual character idle state and the next immediate interaction response to the virtual environment, such as “open door action”; and (3) reaction time at interactive events, such as avoidance of moving obstacles.

Finally, to calculate NAV within-domain latency-based IIV, we used timed response performance data from the following categories, eliciting recruitment of cognitive control processes during DTW: (1) gait velocity during the navigation, measured as the time elapsed between the virtual character idle state and the next immediate “direction command”; (2) cadence, measured as steps per minute between the virtual character idle state and the next immediate interaction response to the virtual environment, such as “open door action”; and (3) time in double support during interactive events, such as avoidance of distractors and moving obstacles.

Following the work described in recent studies (Nesselroade and Salthouse, 2004; Kälin et al., 2014), we calculated the intra-individual standard deviation (ISD) across each individual’s performance profile data in order to compute IIV. Starting from the accuracy-based IIV and the HCS group, we log-transformed performance data in order to achieve normal distribution and multiplied by -1 to adjust for scaling difference. This process generated for HCS standardized residuals representing adjusted accuracy and latency scores with a mean of 0 and variance of ∼ 1. We used the General Linear Model to estimate effects associated with age, education, gender, and potential interactions. The model parameters were used to predict accuracy scores in aMCI and early AD subjects. We then calculated standardized residuals for aMCI and early AD subtracting the predicted from the observed accuracy scores and dividing it by the model’s SE. Similar to the work of Kälin et al. (2014), we used the intra-individual mean (IIM) across residuals underlying across-domain IIV (across-domain IIM) and across residuals underlying within-domain IIV (within-domain IIM) as covariates in all relevant analyses in order to address the association between ISD and mean performance.

Accordingly, for the latency-based IIV we followed data preparation procedures similar to those of Bielak et al. (2010). We removed the high and low outliers in reaction time from each performance profile category for each participant. We defined high outliers as the individual reaction times that were greater than 3 SD more than the person’s mean reaction time and low outliers as individual reaction times less than 3 s. After the outliers were removed, we recalculated mean RT and within-person ISDs for each participant. In order to remove the effect of mean RT from the ISDs, since mean RT is positively associated with variability, and age is associated with slower reaction times (Anstey et al., 2007), we regressed the ISDs on mean RT and collected standardized ISD residuals. Finally, the ISDs of all variables were normally distributed, and we calculated the across-domain IIV and within-domain IIV composite scores of the standardized residuals.

### Genotyping Data

We used restriction isotyping to classify participants as either carriers (APOE ε2/ε4, ε3/ε4, and ε4/ε4) or non-carriers of the APOE ε4 allele. A similar approach is also described in another IIV study (Kälin et al., 2014).

### Statistics

All analyses were performed as two-tailed tests using the statistical analysis software package PASW 18.0 for Windows. We used univariate analysis of variance (ANOVA) to perform the group comparisons of normally distributed demographic, raw, and adjusted performance data applying Sidak *post hoc* tests correcting for multiple comparisons. Not normally distributed variables were analyzed with Kruskal–Wallis tests followed by Mann–Whitney tests corrected for multiple comparisons, and categorical variables were analyzed with Pearson’s chi-square test. Difference in across- and within-domain IIV was analyzed with univariate analyses of covariance (ANCOVA) in order to evaluate group-wise differences with the diagnostic group treated as the main effect. The influences of age, gender, and education as well as across- and within-domain IIM were also used as covariates to control for influences on IIV. To calculate the effect of the IIV type (accuracy- vs. latency-based), we used multivariate analyses of covariance (MANCOVA), and to account for the unbalanced designed we applied Sum of Square Type III. We calculated significant group effects using a Sidak *post hoc* test correcting for multiple comparisons. Finally, all parametric analyses were performed with a significance level of *p* < 0.05, while a significance level of *p* < 0.017 (0.05/3 = 0.017) was applied for non-parametric analyses.

Following a recent study (Montero-Odasso et al., 2014), gait variability was calculated as the coefficient of variation for stride time: CV = (SD of stride time/mean stride time) × 100. Gait velocity (cm/s) and stride time variability (CV_st_, %) were measured during the NAV dual-task trials.

We assessed the test–retest reliability with the intraclass correlation coefficient (ICC) as defined by Shrout and Fleiss (1979). This form of ICC utilizes a two-way ANOVA in which both the SG performance data and participants are treated as random effects to assess reliability at a single point in time. Using this model, test–retest reliability was characterized as excellent (ICC N 0.8), good (ICC 0.6–0.79), moderate (ICC 0.4–0.59), fair (ICC 0.2–0.39), or poor (ICC b 0.2; Kam et al., 2012). We assessed the stability of ICC using three SG data collection sessions by calculating the ICC values from the first two sessions and comparing them to the values from all three sessions.

## Results

The baseline demographic details are summarized in **Table 1**, including IIV computations from the baseline neuropsychological performance.

**Table 1:** Clinical characteristics of the subjects (means with SDs).

**Population**					
*N*		227			
Subgroups		**HCS**	**aMCI**		**Early AD**
*n*		76	65		86
Age (years)		70.06 (13.32)	72.63 (10.05)		76.59 (10.58)
Female		38 (65%)	40 (62%)		54 (63%)
Education		16.1 (6.4)	15.6 (8.0)		8.6 (5.6)
**Neuropsychological data**			
Global	MMSE	29.1 (0.6)^∗∗^	27.1 (0.8)^∗∗^		22.3 (4.2)^∗∗^
Memory	Free recall†	43.2 (9.1)^∗∗^	35.6 (10.9)^∗∗^		22.2 (10.8)^∗∗^
	RAVLT delayed recall	12.7 (1.8)^∗∗^	11.0 (3.1)^∗∗^		9.3 (10.4)^∗^
	Digit span forward	6.0 (2.1)^∗∗^	5.0 (2.1)^∗∗^		3.0 (1.9)^∗∗^
Attention	Stroop trial 3	47.5 (11.1)^∗^	42.1 (13.4)^∗∗^		28.6 (15.3)^∗∗^
	TMTB	140.4 (53.3)^∗∗^	198.4 (91.9)^∗∗^		220.5 (204.2)^∗∗^
	Letter fluency	10.5 (2.9)^∗∗^	8.8 (3.4)^∗∗^		5.6 (2.8)^∗∗^
	Category fluency	19.3 (2.9)^∗^	18.8 (2.2)^∗∗^		16.5 (2.8)^∗∗^
**Psychomotor data**			
Gait speed, m/s	Combined	0.95 (0.2)	0.92 (0.2)		0.88 (0.2)
	Women	0.94 (0.2)	0.83 (0.0)		0.77 (0.1)
	Men	1.02 (0.1)	1.01 (0.0)		0.95 (0.6)
Tapping speed dominant, taps/second	Combined	5.88 (0.8)	5.76 (0.8)		5.74 (0.9)
	Women	5.53 (0.7)	5.49 (0.8)		5.43 (0.7)
	Men	6.29 (0.7)	6.21 (0.7)		6.19 (0.7)
Tapping speed non-dominant, taps/second	Combined	5.63 (0.6)	5.61 (0.7)		3.58 (0.7)
	Women	5.41 (0.5)	5.38 (0.6)		5.33 (0.6)
	Men	5.91 (0.6)	5.96 (0.6)		5.90 (0.6)
**Baseline gait performance**			
Velocity, cm/s	Simple gait	112.9 (15.6)^∗^	96.5 (25.1)^∗^		89.5 (30.4)^∗^
	Counting gait	107.1 (20.7)^∗^	89.3 (22.4)^∗∗^		70.3 (25.5)^∗∗^
	Naming animals gait	98.3 (23.3)^∗^	78.6 (23.3)^∗∗^		69.1 (21.3)^∗∗^
Stride time variability (CV, %)	Simple gait	2.34 (1.3)^∗^	3.53 (2.3)^∗^		4.11 (2.6)^∗^
	Counting gait	2.80 (0.6)^∗∗^	4.90 (3.4)^∗∗^		6.81 (3.3)^∗∗^
	Naming animals gait	3.99 (2.1)^∗∗^	5.73 (6.0)^∗∗^		7.65 (5.0)^∗∗^
**Neuropsychological data IIV**			
Across-domain	IIM	0.00 (0.6)^∗∗^	-1.41 (0.9)^∗∗^		-2.34 (0.8)^∗∗^
Within-domain	IIM	0.01 (0.9)^∗∗^	-0.53 (0.9)^∗∗^		-1.80 (1.0)^∗∗^
Across-domain	IIV‡	0.92 (0.6)^∗^	0.96 (0.6)^∗^		1.37 (0.8)^∗∗^
Within-domain	IIV‡	0.81 (0.6)^∗∗^	1.12 (0.6)^∗^		1.25 (0.7)^∗∗^

† Free recall: sum of the three free recalls.

‡Analyses of covariance, means adjusted for gender, age, and education and across- or within-domain IIM.

^∗^*p* < 0.05, ^∗∗^*p* < 0.001.

*HCS, healthy control subjects; MCI, mild cognitive impairment; AD, Alzheimer’s disease; MMSE, mini-mental state examination; TMT, trail making test; IIM, intra-individual mean; IIV, intra-individual variability*.

In general we observed a main effect between diagnostic groups and IIV [*F*_(2,225)_ = 7.87; *p* = 0.001; η^2^ = 0.07]. We also observed that IIV in general was influenced by age [*F*_(1,225)_ = 4.21; *p* = 0.03; η^2^ = 0.03] and by IIM, although the effect size was not significant [*F*_(1,225)_ = 3.63; *p* = 0.06; η^2^ = 0.02], but not by education [*F*(_1,225)_ = 0.12; *p* = 0.72; η^2^ = 0.00] or gender [*F*_(1,225)_ = 1.78; *p* = 0.27; η^2^ = 0.007]. Groups did not differ in years of education (*F*_(2,225)_ = 1.30, *p* = 0.33) or distribution of gender [χ^2^_(2)_ = 0.29, *p* = 0.89]. HCS and aMCI did not differ in age [*t*_(225)_ = -1.36, *p* = 0.36].

### Accuracy-Based Comparisons Between Across-Domain and Within-Domain IIV

**FIGURE 2:**
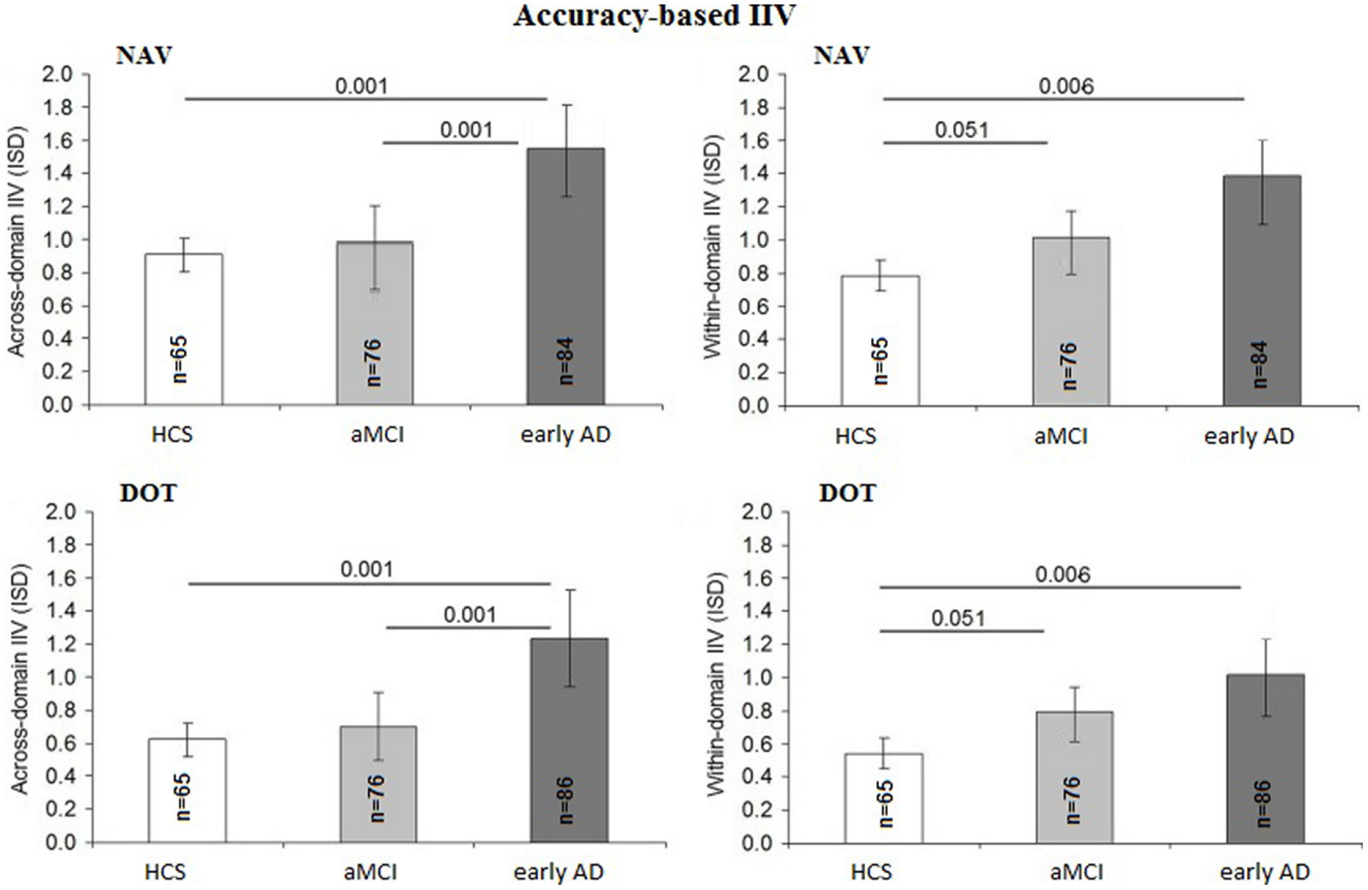
Comparison of accuracy-based intra-individual variability (IIV) scores between diagnostic groups: intra-individual standard deviation (ISD) representing mean across-domain IIV for DOT and NAV as well as mean within-domain IIV for DOT and NAV per diagnostic group (HCS, healthy control subjects; MCI, mild cognitive impairment; AD, Alzheimer’s disease). Error bars display 95% confidence interval for the mean with p values based on Sidak *post hoc* tests following analyses of covariance for the comparison of means adjusted for age, years of education and gender as well as mean across-domain performance **(A)** and mean within-domain performance **(B)**, respectively.

The accuracy-based IIV at both DOT and NAV was not influenced by education [*F*_(1,225)_ = 2.41; *p* = 0.13; η^2^ = 0.01), gender [*F*(_1,225)_ = 2.12; *p* = 0.10; η^2^ = 0.01], or IIM [*F_(_*_1,225)_ = 1.20; *p* = 0.19; η^2^ = 0.01] but differed among diagnostic groups [*F*_(2,225)_ = 5.75; *p* = 0.001; η^2^ = 0.06] and slightly by age [*F*_(1,225)_ = 1.06; *p* = 0.05; η^2^ = 0.02]. The early AD group revealed in general higher across-domain IIV than both the aMCI group (*p* = 0.001; 95% CI = 0.17–0.88) and HCS group (*p* = 0.001; 95% CI = 0.18–0.99), whereas IIV did not differ significantly between the aMCI and HCS groups (*p* = 0.71; 95% CI = –0.16–0.31). Within-domain IIV was not influenced by age [*F*_(1,225)_ = 0.056; *p* = 0.82; η^2^ = 0.00] but was higher in the early AD group compared to the HCS group (*p* = 0.006; 95% CI = 0.12–0.85) and was not significant between the early AD and the aMCI groups (*p* = 0.374; 95% CI = 0.142–0.582). In addition, we found a strong trend for higher within-domain IIV in the aMCI group than in the HCS group (*p* = 0.051; 95% CI = –0.02–0.27). To avoid statistical issues associated with two missing trials for the DOT group, group-level ISD values were imputed for missing data (<3% of the total data). **Figure 2** summarizes the accuracy-based IIV results.

### Latency-Based Comparisons between Across-Domain and Within-Domain IIV

**FIGURE 3:**
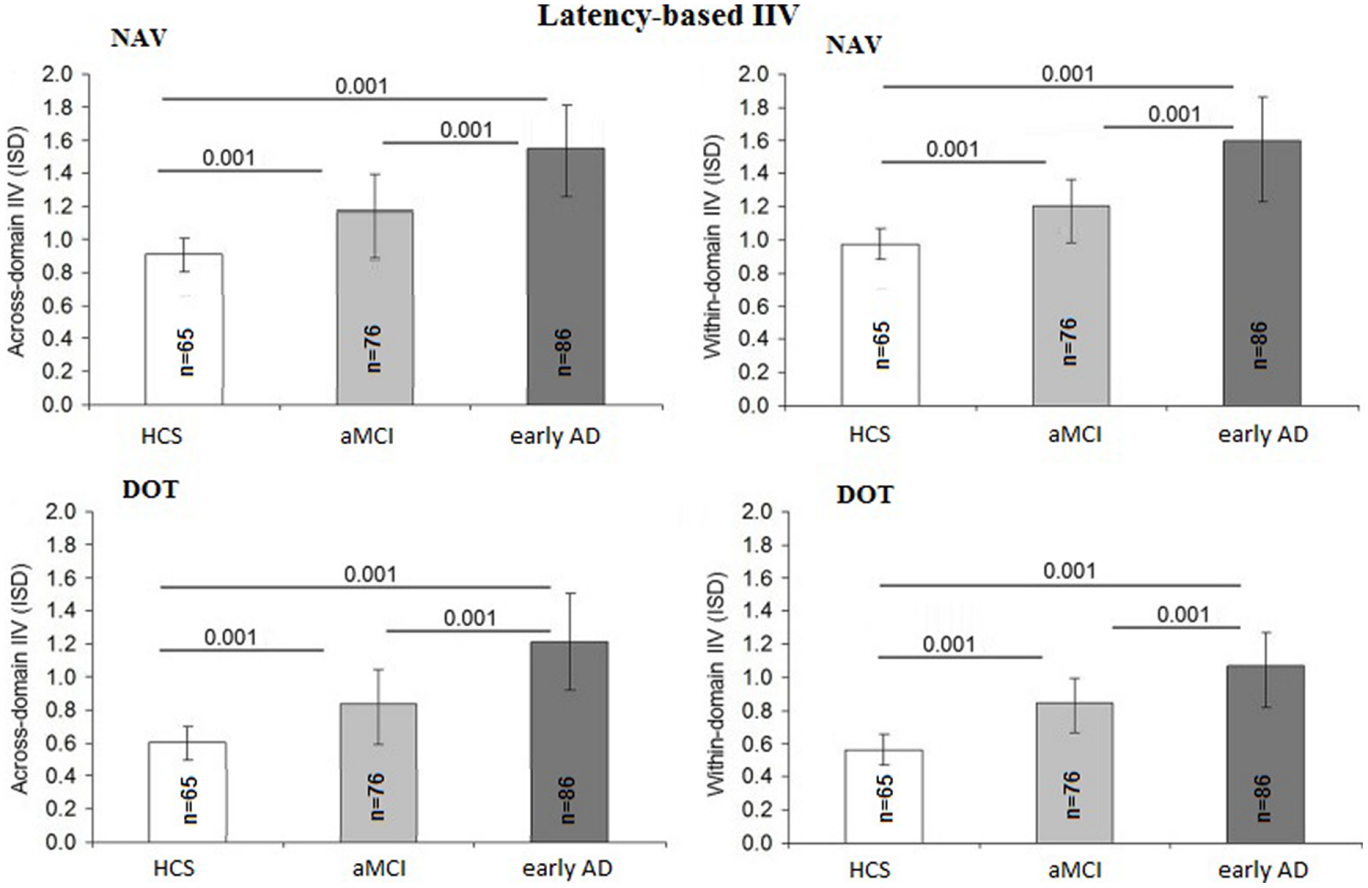
Comparison of latency-based IIV scores between diagnostic groups.

The latency-based IIV at both DOT and NAV was also not influenced by education [*F*_(1,225)_ = 2.43; *p* = 0.15; η^2^ = 0.01], gender [*F*_(1,225)_ = 2.21; *p* = 0.13; η^2^ = 0.01], or IIM [*F_(_*_1,225)_ = 1.19; *p* = 0.20; η^2^ = 0.01] but differed among diagnostic groups [*F*_(2,225)_ = 5.71; *p* = 0.001; η^2^ = 0.06] and age [*F*_(1,225)_ = 1.51; *p* = 0.02; η^2^ = 0.03]. The across-domain latency-based IIV differed between the early AD and aMCI groups (*p* = 0.001; 95% CI = 0.22–1.07) as well as between the AD and HCS groups (*p* = 0.001; 95% CI = 0.24–1.29) and also between the aMCI and HCS groups (*p* = 0.001; 95% CI = 0.26–1.30). Within-domain latency IIV was found to be higher in the early AD group compared to the aMCI group (*p* = 0.001; 95% CI = 0.19–0.85) and the HCS group (*p* = 0.001; 95% CI = 0.16–0.82) and also between the aMCI and HCS groups (*p* = 0.001; 95% CI = 0.20–0.97). **Figure 3** summarizes the accuracy-based IIV results.

### Accuracy-Based IIV vs. Latency-Based IIV

In order to evaluate whether there was a relationship between the accuracy- and latency-based IIV scores and the SG task, a MANCOVA was performed with IIV type (accuracy- or latency-based) and task (DOT and NAV) included as covariates. There was a significant main effect of the IIV type for the group [*F*_(2,225)_ = 29.9; *p* = 0.001; η^2^ = 0.41], but not for task, age, education, or gender.

In addition, ANCOVA was performed in order to calculate a difference score by subtracting within- from across-domain IIV while treating age, education, and gender as covariates. For the accuracy-based IIV, we found only a tendency toward higher across-domain IIV—not within-domain IIV—in each group (HCS: M = 0.21, SD = 0.76; MCI: M = 0.08, SD = 0.81; AD: M = 0.38, SD = 0.95). On the other hand, the latency-based IIV revealed no significant differences between IIV scores across diagnostic groups [*F*_(2,225)_ = 4.32; *p* = 0.16; η^2^ = 0.15].

### Associations between IIV, Genotype, and Neuropsychological Tests

Correlations are given in **Table 2**. The relationship between IIV, neuropsychological tests, and APOE status was explored in a subsample with available genotypes. We performed ANCOVAs to compare IIV scores between ε4 carriers and non-carriers within each group by treating gender as a covariate in aMCI. We calculated the general across-domain IIV and found that it did not vary with APOE status in HCS [*F*_(1,75)_ = 0.412; *p* = 0.37; η^2^ = 0.003], MCI [*F*_(1,51)_ = 0.316; *p* = 0.54; η^2^ = 0.008], or AD [*F*_(1,33)_ = 0.012; *p* = 0.87; η^2^ = 0.00]. Similarly, the within-domain IIV did not vary as a function of APOE status in early AD [*F*_(1,33)_ = 0.219; *p* = 0.67; η^2^ = 0.01], but there was a significant effect of APOE status in HCS [*F*_(1,75)_ = 4.393; *p* = 0.04; η^2^ = 0.04] and aMCI [*F*_(1,51)_ = 2.399; *p* = 0.05; η^2^ = 0.03], which indicated increased within-domain IIV in these groups.

**Table 2:** Cognitive measures per diagnostic group, IIV, and APOE genotype (means with SDs).

**Population**						
*N*		161
Subgroups		**HCS**		**aMCI**		**Early AD**
*n*		16	60	34	18	21	12
APOE status		ε4+	ε4-	ε4+	ε4-	ε4+	ε4-
Age (years)		72.6 (12.02)	70.1 (13.21)	74.1 (9.21)	72.7 (9.44)	76.5 (8.83)	77.8 (9.36)
Male		7 (43.7%)	22 (36.6%)	12 (35%)	7 (38.8%)	8 (38%)	6 (50%)
Female		9 (56.3%)	38 (63.4%)	22 (65%)	11 (61.2%)	13 (62%)	6 (50%)
Education		15.1 (5.9)	15.3 (6.0)	14.5 (7.1)	15.1 (7.4)	8.7 (6.0)	9.1 (5.2)
**Neuropsychological data**
Global	MMSE	29.3 (1.0)	28.9 (0.8)	26.8 (1.9)	27.3 (1.7)	22.1 (3.5)	22.7 (3.2)
Memory	Free recall†	0.02 (1.1)	-0.01 (1.1)	-0.59 (0.6)	-0.62 (0.7)	-0.49 (1.3)	-0.45 (1.0)
	RAVLT delayed recall†	-0.08 (1.0)	0.06 (0.9)	-2.23 (1.6)	-2.14 (1.5)	-3.88 (1.6)	-3.79 (1.4)
	Digit span forward†	0.35 (1.0)	-0.09 (1.0)	-1.43 (1.0)	-0.99 (1.1)	-2.35 (1.5)	-2.59 (1.1)
Attention	Stroop trial 3†	0.03 (0.9)	0.08 (1.1)	-0.69 (0.9)	-0.37 (1.1)	-2.83 (2.4)	-2.99 (2.0)
	TMTB†	0.61 (1.2)^∗^	-0.08 (1.2)^∗^	0.75 (1.3)^∗^	-0.22 (1.3)^∗^	-1.27 (1.6)	-0.36 (1.5)
	Letter fluency†	0.59 (0.9)^∗^	-0.11 (1.1)^∗^	0.67 (1.1)^∗^	-0.25 (1.4)^∗^	-1.32 (1.5)	-0.24 (1.3)
	Category fluency†	0.57 (1.0)^∗^	-0.09 (1.0)^∗^	0.69 (1.2)^∗^	-0.19 (1.3)^∗^	-1.39 (1.3)	-0.49 (1.3)
**IIV**
Across-domain	IIM	0.09 (0.6)	0.01 (0.6)	-1.39 (0.7)	-1.24 (0.6)	-2.38 (1.2)	-2.67 (0.9)
Within-domain	IIM	0.33 (0.7)	-0.10 (0.8)	-0.49 (0.5)	-0.56 (0.9)	-1.91 (1.1)	-1.56 (1.0)
Across-domain	IIV‡	0.81 (0.5)	0.77 (0.6)	1.09 (0.7)	1.23 (0.2)	1.79 (0.6)	1.86 (0.8)
Within-domain	IIV‡	0.56 (0.5)^∗^	-0.18 (0.5)^∗^	0.57 (0.4)^∗^	-0.22 (0.5)^∗^	1.35 (0.8)	1.48 (1.1)

*† Data shown are calculated standardized residuals of cognitive test scores for MCI and AD by using parameter estimates for age, education, and gender in HCS*.

*‡Analyses of covariance, means adjusted for gender, age, education, and across- or within-domain IIM*.

*^∗^*p* < 0.05*.

*HCSs, healthy control subjects; MCI, mild cognitive impairment; AD, Alzheimer’s disease; MMSE, mini–mental state examination; TMT, trail making test; IIM, intra-individual mean; IIV, intra-individual variability*.

### Reliability of Performance-Based IIV Data

**FIGURE 4:**
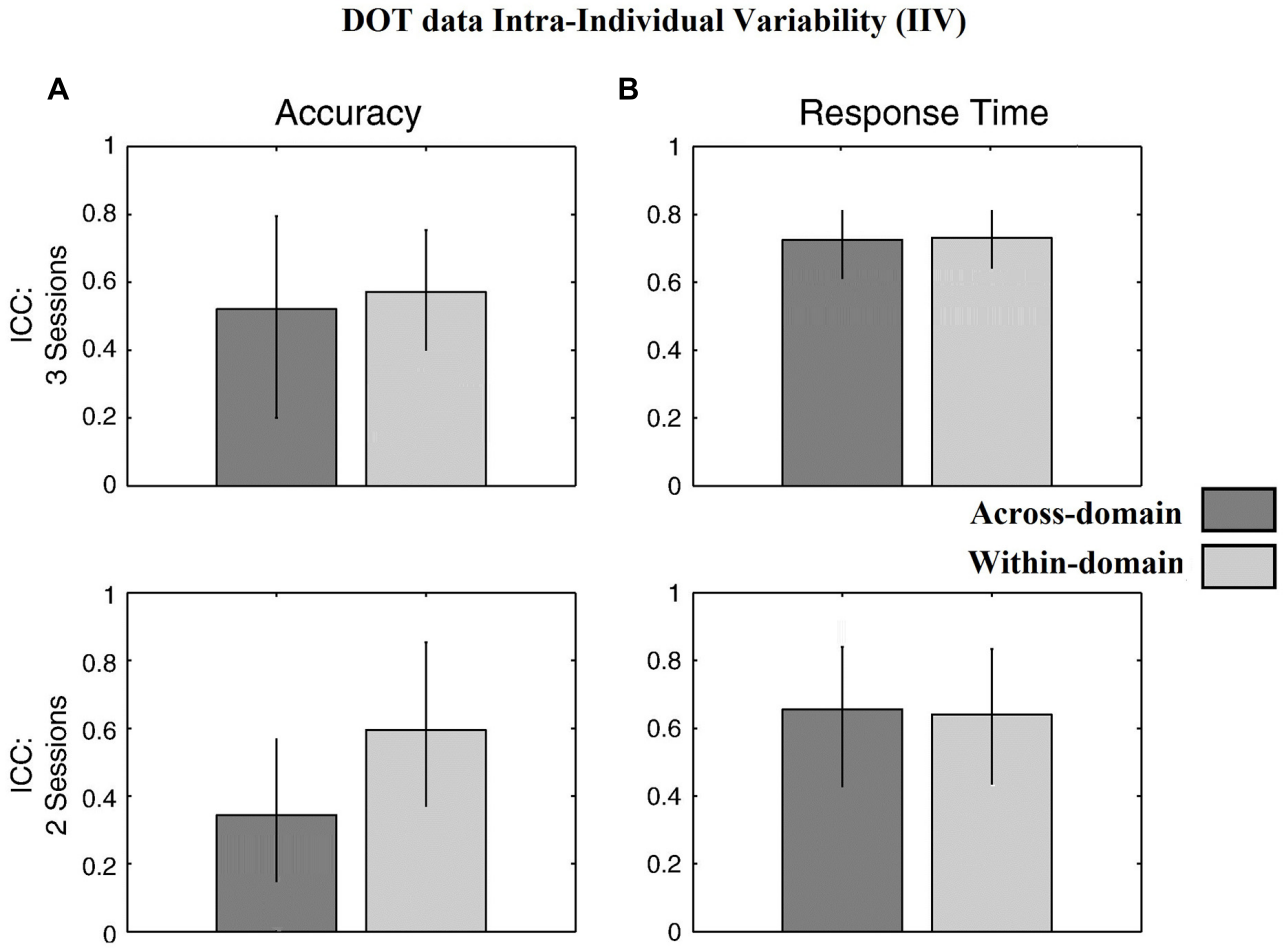
Behavioral results for the DOT task. ICC values from **(A)** accuracy-based and **(B)** latency-based IIV times with 3 and 2 experimental sessions. Error bars represent 95% confidence interval.

**FIGURE 5:**
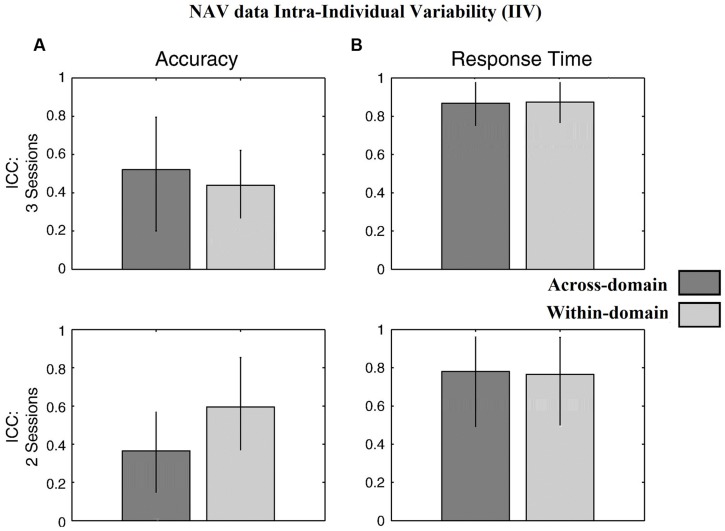
Behavioral results for the NAV task. ICC values from **(A)** accuracy-based and **(B)** latency-based IIV times with 3 and 2 experimental sessions. Error bars represent 95% confidence interval.

**Figure 4** for the DOT task and **Figure 5** for the NAV task summarize the reliability data. ICC values were calculated using all three SG sessions for both the accuracy- and latency-based IIV of the DOT and NAV tasks. Results showed that, in general, accuracy-based IIV elicits fair to moderate reliability (ICC 0.33–0.57) for both the DOT and NAV tasks. In addition, accuracy-based ICC values increased after all three SG sessions for the DOT task, but the NAV task elicited an average ICC decrease of 0.15 (SEM = 0.04). In contrast, latency-based IIV for both the DOT and NAV tasks exhibited good to excellent reliability measures (ICC 0.69–0.85) after all sessions.

## Discussion

A major problem in studying aging is how to separate the effects of aging from disease, and the two most pressing clinical questions relate to etiology and prognosis. In this study, we examined SG with and without DTW performance data and applied across- and within-domain accuracy-based and latency-based IIV measures in order to reliably differentiate between HCS, aMCI, and early AD. Specifically for the SG with DTW, we found increased IIV for both across- and within-domain IIV in early AD vs. HCS, aMCI vs. HSC, and in early AD vs. aMCI, consistent with a recent study (Phillips et al., 2013). In addition, and consistent with the literature on within-domain IIV (Duchek et al., 2009; Schroeter et al., 2012) placing more demands on cognitive control processes, we also found SG with DTW latency-based within-domain IIV being increased in early AD vs. HCS, aMCI vs. HCS, and early AD vs. aMCI, which is found to be a sensitive early marker of cognitive impairment (MacDonald et al., 2012; Kälin et al., 2014). High IIV has been linked to an increased probability that an individual with aMCI will become demented within 2.5 years (Tales et al., 2012).

When one investigates SG-based human navigation—in particular, the strategies implemented in a complex everyday way-finding task—one must also consider the role of vision in walking. In order to maintain a regular walking velocity in the SG, subjects must anticipate visualizing the destination route and process the responses upstream without having to stop. During the NAV task, each way-point had several action points as well as distractors and required several hesitant jerky movements to process them. Using the IIV measures, we were able to verify that NAV task interference is preserved in dual-task conditions, consistent with other recent studies, i.e., (Perrochon et al., 2013).

The role of vision in gait control during locomotion has been demonstrated by other studies, especially when the environment is enriched with visual information (Chapman and Hollands, 2006, 2007). During NAV, this mechanism triggered a modification in gait parameters, such as a reduction in velocity and frequency, and more so by an increase in double support time. At the same time, others have suggested that elders with reduced cognitive ability have more difficulty identifying the environment, and it is necessary to fixate more to have a maximum of visual information (Di Fabio et al., 2005). In another study, Scherder et al. (2011) demonstrated that one can predict a change in walking control and motor performance in subgroups of patients with dementia pathology using the concept of “last in-first out,” —that is, the neuronal circuits that mature late would be the first to deteriorate in neurodegenerative pathology. In that study, subjects with frontotemporal or vascular dementia had difficulties at the motor level in coordinating complex foot movements and planning movements associated with early degeneration of the anterior cingulate cortex and dorsolateral prefrontal cortex. In another study, Gwin et al. (2011) performed an electroencephalogram on a subject walking on DTW and noted activation of the anterior cingulate cortex during placement of the foot, similar to the detection of an error in placing the foot on the floor and correction of its trajectory. During the NAV task, one could imagine that there is a conflict at the level of the anterior cingulate cortex, which simultaneously manages performance of the cognitive task and correct placement of the foot. This conflict at the level of the anterior cingulate cortex could be increased in early AD because this cerebral zone is often prematurely deteriorated in patients with dementia.

We also examined the stability of measurements. Our results found at both the SG accuracy- and latency-based measures an increased IIV, suggesting a breakdown of cognitive control functions early in prodromal AD. More precisely, across- and within-domain accuracy-based IIV differed between each group, underlying the differences in cognitive control required by the DOT and NAV tasks. Consistent with other studies (Kälin et al., 2014), we also found that accuracy-based within-domain IIV was increased in early AD and aMCI vs. HCS and appeared to constitute a reliable marker for the detection of prodromal AD at the MCI stage. We also found that accuracy-based across-domain IIV was increased in early AD vs. aMCI and HCS and may be used to separate early AD from the aMCI stage.

Furthermore, since higher IIV has been found in tasks requiring cognitive control to be influenced by gender, by task-related processing load and processing speed (MacDonald et al., 2009; Phillips et al., 2013; Kofler et al., 2014), previous studies found that MCI subjects who later converted to dementia were found to have higher IIV than non-converters. Consistent with this study and the literature on latency-based within-domain IIV (Duchek et al., 2009; Schroeter et al., 2012) placing more demands on cognitive control processes, we also found latency-based within-domain IIV being increased in early AD vs. HCS, aMCI vs. HCS, and early AD vs. aMCI.

Additionally, we found increased within-domain IIV in HCS and aMCI ε4 carriers vs. non-carriers, whereas there was no ε4-related change in IIV in the early AD group. Contrary to findings reported by others (Duchek et al., 2009; Kälin et al., 2014) who only found an increased latency-based IIV in a cognitive control task in HCS ε4 carriers vs. non-carriers, we also found increased latency-based IIV in aMCI ε4 carriers vs. non-carriers. One reason for our findings might be that, in contrast to the previous studies, we examined the relationship between accuracy- and latency-based intra-individual differences in trial-to-trial variability. Another reason might be that the SG performance data are sensitive enough to detect subtle changes in IIV at both the HCS and aMCI stages. However, such interpretations should be treated with caution as it is already known that the frontal lobe constitutes a brain region that manifests ε4-effects very early in the disease (Filbey et al., 2010). Since the frontal lobe is believed to be the basis of IIV (MacDonald et al., 2009), our findings add evidence to recent studies (Kälin et al., 2014) and further support the relationship between within-domain IIV and APOE status.

In order to assess the reliability of performance data IIV, we analyzed the stability of test–retest measurements for both the DOT and NAV tasks using ICC values. Given that longitudinal change in IIV among accuracy and response time is thought to be particularly important and robust in signaling the risk of cognitive impairment and dementia (Vaughan et al., 2013), this result underscores the importance of utilizing response time data as a metric for memory processes, especially in aMCI populations where memory decline is often the behavioral outcome of interest.

In summary, our results demonstrated that SG with DTW performance profiles data represents another aspect of cognition that underlies age-related differences in cognitively demanding tasks independently of mean reaction time and executive function. Importantly, our findings confirmed that impaired cognitive control processes, especially in terms of latency, as measured with NAV performance profiles, produce stable inconsistencies across IIV in cognitive control-sensitive tasks, and hence can act as a predictor of greater cognitive decline.

Consistent with other studies, we found intra-individual differences in cognitive domains, both cross-sectional as well as longitudinal, which can be used for early detection and intervention. Recently, evidence for a strong association between IIV and frontal gray and white matter integrity changes on MRI scans (volumetric decline, demyelination, and hyperintensities) due to age-related changes in cerebral bold flow, vascular injury, or neurological conditions such as AD (Jackson et al., 2012; Lövdén et al., 2013; Radanovic et al., 2013) support the idea of frontal system disruptions underlying increased IIV in aMCI and early AD.

Our study has strengths but also limitations. One limitation was the small variability of the education profiles of our groups, which were all considered to be highly educated older adults. Although recent studies (Kälin et al., 2014) found no effect between education and IIV, this risk was addressed by treating education and within- and across-domain IIM as covariates in all analyses. Furthermore, the outcome of interest in the present study was the ISD calculated across tasks, and thus we assume the risk to be minimal. Another limitation was the correlation of SG performance profile data with neuropsychological tasks that might not exclusively assess the same cognitive functions. Specifically, the NAV task required motor performance with complex cognitive abilities, such as processing speed, visuo-construction, and inhibition, among other cognitive control functions. Neuropsychological tasks, such as the TMT, the Letter and Category Fluency task, the Stroop Test, and the RAVLT place fewer demands on cognitive control processes, which might have influenced the significance of our correlations. Finally, the motor performance seen in subjects with aMCI is likely to be linked to early degeneration of the dorsolateral prefrontal cortices as well as the anterior cingulate cortex, and these subjects would be susceptible to progressive dementia pathology, such as frontotemporal and vascular dementia and not only AD. Brain imaging will be used in a future study to confirm or refute this hypothesis.

Despite these limitations, SG with DTW might useful in everyday clinical practice. Compared to other non-invasive biomarkers such as MRI, using SG performance data in clinical practice could optimize the diagnosis of AD at the early stage of the disease and would provide the greatest benefit in terms of cost and risk compared with other techniques. Finally, we suspect that integrating this type of dual-tasking with training programs or physical therapy, in an acute training design, might delay cognitive and motor decline in the elderly. However, further examination using different custom SG tasks in a longitudinal design is needed to provide more specific information about their preventive value.

## Author Contributions

For the work described in this manuscript, IT developed and implemented the DAT and NAV system, performed manuscript drafting and statistical analysis. DK performed data collection and analysis. MT and SP performed the recruitment of participants and drafted the Ethical Approval. IT, BD and MD conceived the study and participated in its design, and coordinated the Grant funding. All authors did read and approve the final manuscript.
